# Compensating unknown speed of sound in learned fast 3D limited-view photoacoustic tomography

**DOI:** 10.1016/j.pacs.2024.100597

**Published:** 2024-02-17

**Authors:** Jenni Poimala, Ben Cox, Andreas Hauptmann

**Affiliations:** a Research Unit of Mathematical Sciences, University of Oulu, Finland; b Department of Medical Physics and Biomedical Engineering, University College London, UK; c Department of Computer Science, University College London, UK

**Keywords:** Photoacoustic tomography, Convolutional neural networks, Complex valued neural networks, Image reconstruction, Speed of sound compensation

## Abstract

Real-time applications in three-dimensional photoacoustic tomography from planar sensors rely on fast reconstruction algorithms that assume the speed of sound (SoS) in the tissue is homogeneous. Moreover, the reconstruction quality depends on the correct choice for the constant SoS. In this study, we discuss the possibility of ameliorating the problem of unknown or heterogeneous SoS distributions by using learned reconstruction methods. This can be done by modelling the uncertainties in the training data. In addition, a correction term can be included in the learned reconstruction method. We investigate the influence of both and while a learned correction component can improve reconstruction quality further, we show that a careful choice of uncertainties in the training data is the primary factor to overcome unknown SoS. We support our findings with simulated and *in vivo* measurements in 3D.

## 1 Introduction

Photoacoustic (PA) imaging is increasingly being used in clinical situations [1], [2] especially where there are vascular changes, such as with some types of cancer or inflammation. In clinical scenarios, PA imaging systems that use linear (1D) or planar (2D) array transducers are popular (see for example [3], [4], [5], [6], [7], [8]). This is because, despite their inherent limited-view, they fit in well with existing clinical workflows: they can be made into small devices, similar to handheld ultrasound imaging probes, and the small amount of data and size of image volume mean that reconstructions can typically be performed with frame rates fast enough for real-time imaging. However, there are two challenges in real-time reconstructions from linear and planar arrays, concerning how to deal with (1) the unknown sound speed [9], and (2) the missing data from the limited-view detection [10]. The failure to use the correct sound speed will result in blurring of the image in the form of a kind-of double-vision artefact in which the measured waves are not mapped back into quite the right place in the image, while the limited-view detection gives rise to blurring perpendicular to the array and characteristic curved artefacts.

*Unknown Speed of Sound*

Because of its central importance to PA imaging, a variety of approaches have been proposed to tackle the problem of the unknown SoS. A spatially-varying SoS could, in principle, be recovered jointly with the PAT reconstruction, although this problem is inherently unstable and requires additional data or prior knowledge [11], [12], [13], [14], [15], [16], [17]. A SoS image could be found using a separate modality, such as ultrasound tomography [18], [19], [20], [21], but this requires additional time-consuming measurements. Furthermore, PA reconstructions that use a spatially-varying SoS in general require a numerical model of the wave equation for heterogeneous media [22], which are not currently fast enough to facilitate real time reconstructions. Computationally faster alternatives assume, for instance, a known two-component SoS-map [23]. Approaches to exclude the data components that have been strongly aberrated by truncating measurements in time have been proposed [24], [25], as has the estimation of the acoustic travel time using correlations [26], but these methods are not applicable to the one-sided measurements of interest here. In the Bayesian setting, errors due to uncertainties in the SoS can be compensated for using Bayesian approximation error modelling [27], but the method is computationally intensive. More practically, methods have been proposed to estimate the SoS automatically, by maximising an image quality metric or similar [28], [29], [30], [31], [32], [33]. However, all of these proposed methods for accounting for SoS heterogeneities in PA reconstruction either require additional hardware, prior knowledge of the target, or cannot be applied in real-time, or are not applicable to the one-sided measurements of interest here.

Recently, advances in learning-based methods for inverse problems and tomographic reconstructions [34], [35], [36], [37], [38] offer the prospect of ameliorating the SoS problem in a data-driven manner. Based on the model-based approach in [11] that jointly reconstructs and estimates SoS maps an extension to a learned iterative version has been proposed in [39]. In [40], the reconstruction operator that takes into account SoS variations is given by a network architecture. In [41] a variable set of SoS has been used to create the training data to train a learned iterative reconstruction. Similarly, in [42], a model correction is performed after the model evaluation i.e. the reconstructed image is corrected by using a network that has learned from different SoS distributions. Recently, the authors in [43] propose to use explicitly one-hot-encoded input SoS to train and evaluate their network.

*Limited-View Detection*

The challenge presented by limited-view detection has been tackled in a variety of ways. For example, by making multiple measurements of a series of initial acoustic pressure distributions patterned such that they emit wavefronts in the direction of the detector [44], [45], by using reflectors to direct the acoustic energy back to the detector array [46], [47], [48], and many papers that use regularisation or a compressed sensing approach, eg. [49], [50], [51], [52], [53], [54]. More recently, there has been an explosion of learned methods proposed to ameliorate the missing data and thereby reduce the related artifacts [55], [56], [57]. These methods can be roughly divided into three categories: learning-based direct reconstruction, learning-based post-processing and model-based learned iterative reconstruction. In the learning-based direct reconstruction, a network is used to reconstruct an image directly from the sensor data. Several different networks have been studied including for example U-Net [58], FPnet [59], Y-Net [60] and Pixel-DL [61]. In the learning-based post-processing reconstruction method, a network is applied after a conventional reconstruction method. Most commonly used network architecture is U-Net [58], [62], [63], [64], but for example DALnet [65] and WGAN-GP [66] have been utilised. Model-based learned iterative reconstructions use a network to learn an iterative update including a learned regularisation following conventional model-based iterative methods [67], [68], [69]. In addition, neural networks have been used to extend limited-view data to full-view data, allowing artefact reduction when conventional reconstruction methods are utilised [70].

*Contribution of This Paper*

In this paper we propose a data-driven approach to ameliorate both the limited-view and unknown SoS problems jointly given suitable training data. In particular, we augment a Fourier domain reconstruction approach from planar measurements with learned components. Image reconstruction in the Fourier domain [71], [72], assuming a constant SoS, is a popular method for PAT reconstruction when the data is measured using a linear or planar array. It is fast, because it exploits the Fast Fourier Transform, and it is exact when the sound speed in known and in the limit of the array extending to infinity. We note, that the developed methodology can be applied to measurements from circular sensor as well, given corresponding fast Fourier reconstruction methods [73], but is outside the scope of this study.

Correction of SoS and limited-view correction cannot be separated. Thus, here a learned version of this Fourier-domain reconstruction is introduced, with two data-trained networks embedded in the standard Fourier reconstruction: one in image *k*-space, the second in the image space itself, which are trained jointly. The use of two networks is motivated by the observation that we know *a priori* that one effect of an incorrect SoS will be on the mapping from the temporal frequency to the spatial frequency domain, suggesting a network in *k*-space. We also know that the limited-view geometry will lead to curved streak-like artifacts, which motivates the use of a post-processing network in image space, known to facilitate the removal of such artifacts.

We then systematically evaluate the influence of the model correction and training data selection on simulated data with unknown homogeneous and heterogeneous SoS distributions, as well as on *in vivo* data. We show that while a model correction term can improve reconstructions quality, ensuring that the uncertainties in SoS are well-captured in the training data is crucial. Consequently, this study provides further insights into learned reconstructions with (partially) unknown SoS maps by providing a systematic evaluation of training data selection and the potential to learn a joint correction in image as well as image *k*-space.

## 2 Methods

### 2.1 Photoacoustic model and reconstruction

In photoacoustic tomography the acoustic wave propagation is modelled as an initial value problem for the acoustic wave equation. That is, given the initial acoustic pressure $p_{0}$ inside the tissue domain and a spatially dependent sound speed c(x), neglecting attenuation and shear waves, we consider

(1) $$\left(\partial_{tt}-c{\left(x\right)}^{2}\Delta \right)p\left(x,t\right)=0,\;p\left(x,0\right)=p_{0}\left(x\right),\;\partial_{t}p\left(x,0\right)=0.$$

In our application, the measured pressure wave p(x,t) is recorded on the boundary of the domain, here given as a planar sensor. The measured time-series is then modelled by a filtering and mapping operator to the boundary as

(2) g=Mp+ϵ,

with measurement noise ϵ. Eq. (2) will be used in this study as our measurement model for the forward problem. We note that subsampling may be applied and included in M as well, but it is omitted in this study. Given the measured time-series g one could compute a reconstruction of $p_{0}$ by time reversal using full wave solvers [74]. This allows heterogeneous SoS distributions to be included, but is computationally expensive, especially in three dimensions. Alternatively, many fast solvers have been proposed over the years, most notably for a planar detection geometry there are fast Fourier transform based methods available [71], [72], [75]. Unfortunately, these efficient methods are based on the assumption of constant homogeneous SoS. The subject of study here will be how to include a data-driven correction to compensate for heterogeneous or unknown SoS, while maintaining the computational efficiency.

The reconstruction algorithm under consideration here follows [71] and assumes constant SoS $c_{0}$ inside the tissue and a planar detection surface, then $p_{0}$ can be obtained from the two-dimensional measurement $g\left(x_{1},x_{2},t\right)$ via

(3) $$\overset{˜}{f}\left(k_{1},k_{2},\omega \right)=B\left(k_{1},k_{2},\omega \right)F_{1,2}\left(C_{t}\left(g\left(x_{1},x_{2},t\right)\right)\right)$$

(4) $${\overset{˜}{p}}_{0}\left(x\right)=F_{k}^{-1}\left(\overset{˜}{f}\left(k\right)\right)$$

where $\overset{\sim }{f}\left(k_{1},k_{2},\omega \right)$ is obtained from $\overset{˜}{f}\left(k\right)$ via the dispersion relation ${\left(\omega /c_{0}\right)}^{2}=k_{x}^{2}+k_{y}^{2}+k_{z}^{2}$ and F, C denote the Fourier and Cosine transform on the respective variables. The weighting factor B is given by,

(5) $$B\left(k_{1},k_{2},\omega \right)=\left(\sqrt{{\left(\omega /c_{0}\right)}^{2}-k_{x}^{2}-k_{y}^{2}}\right)/\omega .$$

Thus, a reconstruction of the initial pressure $p_{0}$ can be efficiently computed by applying the FFT on the detection surface, a multiplication in *k*-space, an interpolation, followed by an inverse FFT. Nevertheless, this procedure comes with two major limitations, namely the assumption of constant SoS as well as the restriction to planar detection surfaces. In the following we will propose one way to augment the inversion algorithm in Eqs. (3), (4), such that these limitations could be overcome by learning a correction in image *k*-space as well as image space.

### 2.2 Introducing a learnable SoS correction

Even though the FFT reconstruction assumes some simplifications in the model, it is desirable to use due to its efficient implementation. The aim in the following is to correct for some of the simplifying assumptions. In particular, in the constant case if the SoS is not known a wrong choice of SoS can lead to severe reconstruction artefacts and thus SoS would need tuning to obtain the best imaging quality [28], [29], [32]. A more severe limitation is the lack of handling heterogeneous SoS distributions. To implement the correction, we note that the SoS $c_{0}$ is involved in the weighting factor and the interpolation from Eq. (3) to Eq. (4). Thus, it would be natural to either include a step before or after the interpolation to the k-grid to compensate for the inaccurate assumption. Here, we chose to perform the interpolation first and then correct potential errors. For this purpose we introduce a convolutional neural network $G_{\psi }$ in the reconstruction process, such that the full reconstruction becomes

(6) $$\overset{˜}{f}\left(k_{1},k_{2},\omega \right)=B\left(k_{1},k_{2},\omega \right)F_{1,2}\left(C_{t}\left(g\left(x_{1},x_{2},t\right)\right)\right)$$

(7) $$\overset{˜}{f}\left(k_{1},k_{2},\omega \right)⟶\overset{˜}{f}\left(k\right)$$

(8) $$f\left(k\right)=G_{\psi }\left(\overset{˜}{f}\left(k\right)\right)$$

(9) $${\overset{˜}{p}}_{0}\left(x\right)=F_{k}^{-1}\left(f\right).$$

We will denote the above learned reconstruction procedure Eqs. (6)–(9) by $F_{\psi }^{†}$, with the learnable network parameters ψ. That is, we obtain the reconstruction ${\overset{˜}{p}}_{0}$ from the measured data g as ${\overset{˜}{p}}_{0}=F_{\psi }^{†}\left(g\right)$, after training as we will discuss below. The purpose of the correction network $G_{\psi }$ is to locally adjust the misaligned frequency information to its correct form. This misalignement is caused by using the wrong SoS to define the mapping from temporal frequency to spatial frequency and in the weighting factor B. Since the network $G_{\psi }$ corrects for incorrect model assumptions in the inversion process, we will refer to it as a model correction in the following.

Finally, we point out that the network $G_{\psi }$ operates here in *k*-space and as such works on complex data. There are two ways to achieve this, either separating real and imaginary parts and train separate networks or utilising complex convolutional neural networks. In the former, the interaction between real and complex part is lost, consequently it is natural to consider networks that operate on the complex values as described below. Additionally, it has been reported in the literature that improved performance can be achieved by using complex valued networks on *k*-space data in magnetic resonance imaging [76], [77].

#### 2.2.1 Complex convolutional neural networks

In complex convolutional neural networks [78], inputs, weights and activation functions are complex. Let the complex input be h=x+iy where x and y are real vectors. In the complex weight, we allocate the first half of the feature maps to represent the real components and the remaining half to represent the imaginary ones e.g. complex weight is W=A+iB where A and B are real matrices. When convolving the weight W with the input h, the output is given by

W∗h=(A∗x−B∗y)+i(B∗x+A∗y).

Numerous activation functions have been proposed for complex networks, but complex numbers are shown to be especially sensitive to the choice of non-linearity [78]. A good choice for the non-linearity is a complex rectified linear unit (ReLu) due to its good convergence and phase manipulating properties [78], offering a natural equivalent to real valued architectures. This is defined as the complex activation that applies separate ReLU activations on both of the real and the imaginary part of a neuron, i.e

ℂReLU(z)=ReLU(Re(z))+iReLU(Im(z)).

In addition to the activation function, a batch normalisation and weight initialisation are also key components of complex valued networks. Thus, complex valued versions of these algorithms are needed and they may be formed according to the same principles as their real valued counterparts. Similarly, complex equivalents can be found for other components of neural networks as well.

### 2.3 Joint correction and learned reconstructions

The purpose of the correction term in Eq. (8) is primarily to adjust for mismatches in the SoS distribution. The planar scanning geometry will still lead to limited-view artefacts in the reconstruction that the correction alone is not designed to compensate for, as this would require an interpolation task in the *k*-space and is a different learning problem. Here, we rather train the network to shift the *k*-space data to compensate for the wrong SoS, this will be also reflected in the chosen architecture as we discuss below in Section 3.1. Thus, the augmented reconstruction method still needs to be combined with another data-driven reconstruction method to achieve state-of-the-art reconstruction quality by compensating for the limited-view artefacts in the reconstruction space. For this study, we will concentrate on the approach of a post-processing network here to maintain the computational efficiency and potential for real-time applications. That is, given a reconstruction ${\overset{˜}{p}}_{0}$ we train a secondary network $\Lambda_{\theta }$, such that

(10) $$\Lambda_{\theta }\left({\overset{˜}{p}}_{0}\right)\approx p_{0}$$

by minimising a loss function of the form

(11) $$\ell \left({\overset{˜}{p}}_{0};\theta \right)=\|\Lambda_{\theta }\left({\overset{˜}{p}}_{0}\right)-p_{0}\|.$$

The question remains, how the correction network $G_{\psi }$ should be trained, either separate to $\Lambda_{\theta }$ or jointly. In fact, it has been shown in several studies that an end-to-end approach is more beneficial [79], i.e., the whole process should be trained jointly. That means, the mapping now takes in the measurements g directly and the trainable reconstruction operator becomes

(12) $$\Lambda_{\theta }\left(F_{\psi }^{†}\left(g\right)\right)\approx p_{0}$$

with the loss function

(13) $$\ell_{j}\left(g;\theta \right)=\|\Lambda_{\theta }\left(F_{\psi }^{†}\left(g\right)\right)-p_{0}\|.$$

The crucial part for a successfully training of the joint reconstruction operator, is that the derivative with respect to ψ inside the model can be computed (jointly with θ), which in this case only involves the Fourier transform in Eq. (9). To this end, we want to emphasise that the network or any learned component can be included anywhere in the reconstruction procedure in Eqs. (6)–(9), even on the measurement data itself, as long as the gradient can be computed through the procedure for training the networks jointly. We achieve this by providing an implementation that fully supports automatic differentiation as we discuss in the next section.

Finally, training data for the model correction component will be crucial, as the network needs to learn how and what needs to be corrected for. We will discuss the creation and use of training data in the next section.

## 3 In silico and *in vivo* experiments

We use both the numerical simulations and human *in vivo* data to examine the performance of the learned based reconstruction approaches described in Section 2. In addition, time reversal reconstruction and FFT-based reconstruction were computed as a baseline reconstructions.

Accuracy of the reconstructions was evaluated by computing the peak signal to-noise ratio (PSNR) and structural similarity index (SSIM) of the reconstructions with respect to the true initial pressure distribution.

### 3.1 Network implementation

All reconstruction methods and networks were implemented in Python using PyTorch [80]. In the implementation of FFT-based reconstruction, we followed the k-wave implementation [81]. We implemented the FFT reconstruction with native PyTorch functions, for full GPU support and to benefit from the inbuilt automatic differentiation and as such backpropagation can be efficiently computed. The most critical part is the interpolation in Eq. (7) from data *k*-space to image *k*-space, for which we used the RegularGidIterpolator from the torch_interpolations class. The codes are published^1^ along with this study as part of our contribution.

In this work, we considered two different network architectures. For the model correction network $G_{\psi }$ in Eq. (8), we used a very simple network that had three complex convolutional layers. In the first two layers, complex convolution was followed by complex batch normalisation and CReLU. We used a filter size of 3 × 3 and the first two layers had a width of 32 filters. For the implementation of the complex network, we used the complexPyTorch package,^2^ see also [82] for an application.

For the post-processing network $\Lambda_{\theta }$ in Eq. (10), we used a U-Net type architecture [83], [84]. U-Net has demonstrated strong robustness and a diverse applicability to different research fields. The U-Net architecture used here consisted of four scales, i.e. three down and up-sampling layers, with a window size of 2. In each scale, we applied a two convolutional layers followed by batch normalisation and ReLU. Here, we have chosen the same size of all convolutional kernels as 3 × 3 and the width in the first scale 32, which in each downsampling was doubled, that is in the finest scale we used 256 filters.

Finally, let us motivate the choice of architectures for the different tasks. In the model correction the network $G_{\psi }$ primarily needs to correct for the shifted *k*-space data. That means, we do not need a large receptive field as the network should operate locally. Thus, a small complex convolutional network was a good choice. In the image domain, this is different. Due to the limited-view nature we needed a larger receptive field, for which a U-Net is very well suited. We still used a smaller network version of U-Net with only 3 downsamplings instead of 4, as we wanted to preserve some of the non-locality of convolutions.

### 3.2 Computational details

In the computations, a rectangular domain of size 120×120×40 with a discretisation of dx=dy=dz=0.106mm was considered. As a measurement geometry, we consider a plane detector at the top of the domain. The sensor pitch was same as the spatial discretisation in x axis.

A carefully designed training regime has been devised to provide algorithm dependent convergence, which was used for all experiments. In particular, a sufficiently large number of training iterations was needed for the model correction to generalise, we ensured convergence of the training by using PyTorch’s Adam algorithm with a cosine decay. We observed dependence of results based on learning rates and thus we chose 3 different initial learning rates 10^−4^, 10^−3^, 10^−2^. Finally, due to memory limitations a batch size of 1 was used. We note, that we did not use a validation set to choose locally optimal training points, but rather let the training convergence and report the best performing result on the test data. Training of the post-processing network took around 3.5 h, but training of model correction and post-processing networks together took around 12 h. Although training of networks is time consuming, computation of reconstructions after the training is fast. Post-processed reconstruction can be obtained in 0.016 s and model-corrected and post-processed reconstruction takes 0.020 s, we note that this is the full reconstruction time in 3D. All training and image reconstructions were performed using a workstation with a Nvidia Quadro RTX 6000 GPU with 24 GB memory.

In terms of computational complexity, the complex convolutions are four times more expensive to evaluate than real valued convolutions. This can also be seen in the memory consumption of the methods. The post-processing approach consumed around 3.7 GB memory whereas model correction and post-processing consumed around 8.5 GB due to an additional network in image *k*-space, although the neural network of the model correction was much smaller. We note, that larger imaging volumes can be considered and require primarily more memory.

### 3.3 Simulation experiments

**Fig. 1:**
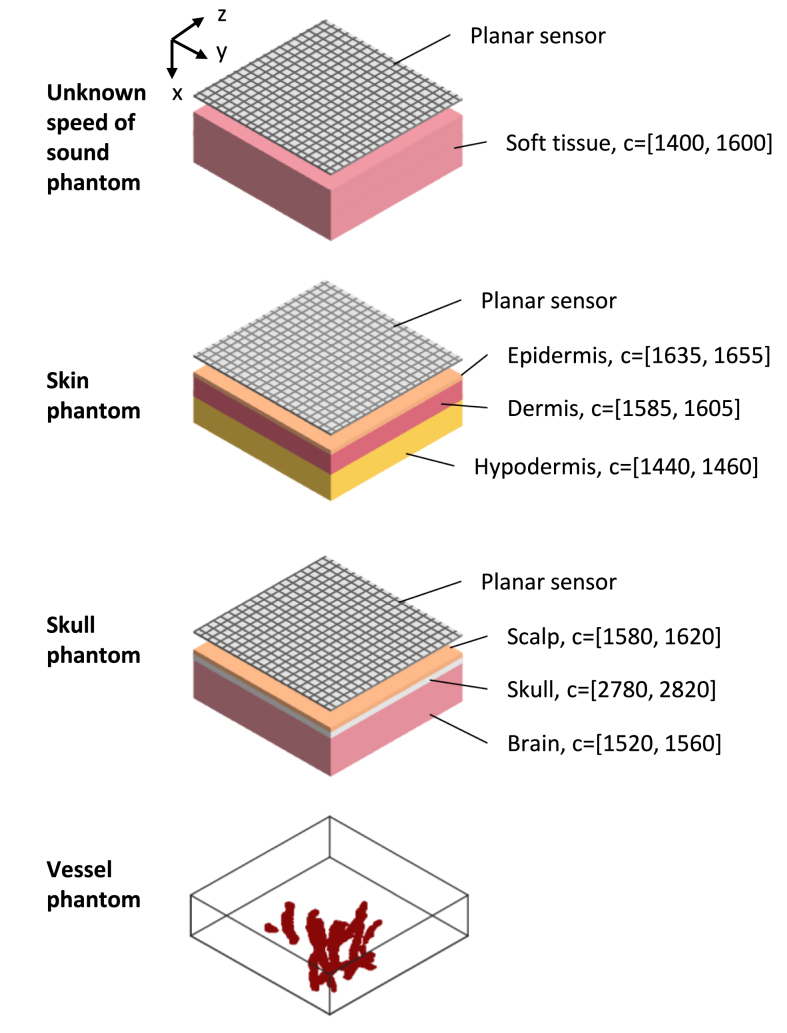
Phantoms used in the simulations. Top three rows show different acoustical properties of the phantoms. In each region, the speed of sound of the voxel is randomly chosen from uniform distribution of the given interval. Bottom row shows an example 3D volume of the vessel mimicking initial pressure distribution. In the case of the skin phantom, parts of the vessel phantom that are located to epidermis were excluded. In the case of the skull phantom, only parts of the vessel phantom that are located in the brain tissue are included. The sensor was in acoustic contact with the tissue.

In the simulations, two problems containing different acoustical properties were considered. In the first problem, a numerical phantom that possessed a constant SoS was studied. In the second problem, two numerical phantoms that consisted of three regions with varying SoS were examined. The considered problems are illustrated in Fig. 1. In the each problem, the true SoS was assumed to be unknown and therefore a commonly chosen constant SoS of 1500m/s for soft tissue imaging was used in the reconstruction. The goal of simulations, especially in the first problem, was not only to compare the performance of reconstruction methods but also to study the impact of the choice of the training data.

#### 3.3.1 Unknown SoS

In PAT we are often interested in imaging vasculature, so we used a numerical vessel mimicking phantom (see Fig. 1) to test approaches. The numerical vessel phantoms were obtained by segmenting vessels from lung CT scans previously used in [85] and smoothing them with a Gaussian filter. These smoothed vessels phantoms were used as ground truth images (true initial pressure distribution) and they were divided to train and test images. Training images included 300 samples and test images included 45 samples. We note that due to the locality of convolutional neural networks and the 3D volume sizes the training data does contain in fact more information than the number of samples suggests. This is in contrast to image classification, where each sample image only maps to a single class. Nevertheless, the U-Net architecture includes down and up-sampling operations, which combined with the nonlinearities allows the network to learn global dependencies necessary for the compensation of limited-view artefacts in the deep tissue area. We note, that the provided sample size, while still of limited size, proved sufficiently large in our training procedure to achieve generalisation to the testing data.

To simulate the corresponding synthetic measurement data we have used the k-Wave MATLAB toolbox [81]. The pairs of ground truth image and synthetic measurement data was collected to form train and test set. In the case of test set, a constant SoS for the whole domain was uniformly randomly chosen from the range of [1400,1600]m/s for each test image. In the case of training, we created three train sets, so that each set had a different range from which a constant SoS was uniformly randomly chosen for each training image. The ranges were [1400]m/s, [1500]m/s and [1400,1600]m/s. For all sets, the pressure signals were recorded for 200 time steps at a temporal sampling rate of 20MHz and a Gaussian noise with a standard deviation of 1% of the maximum value of the synthetic measurement data was added to the simulated pressure signals. In the reconstruction, a fixed constant value for the SoS of 1500m/s was used.

#### 3.3.2 Heterogeneous SoS distribution

In practice, the SoS of tissue is rarely constant. Thus, we also considered two phantoms that have heterogeneous SoS distributions. The phantoms included three layers with flat boundaries and a varying SoS. Although this is a simplistic model for real cases, we consider it sufficient because variations in the speed of sound cause similar phase variations as irregularly shaped boundaries.

The first phantom was a numerical skin phantom. The first layer was an epidermis of thickness 0.212mm and the SoS values were on the interval [1635,1655]m/s. The second layer was a dermis of thickness 2.12mm and the SoS values were on the interval [1585,1605]m/s. The third layer was a hypodermis of thickness 1.696mm and the SoS values were on the interval [1440,1460]m/s. The initial pressure distribution was again the same vessel phantom. This time the phantom was zero-filled so that no nonzero values were located on the epidermis after the smoothing.

The second phantom was a numerical skull phantom. Three layers were skin, skull and brain, respectively. The skin layer had a thickness of 0.212mm and the SoS values were on the interval [1580,1620]m/s. The skull had a thickness of 0.428mm and the SoS values were on the interval [2780,2820]m/s. In this simple skull model, shear effects and scattering from the diploë were not considered. The rest of the domain was considered to be brain and the SoS values were on the interval [1520,1560]m/s. Also in this case, the same vessel phantom was used as the initial pressure distribution, but this time nonzero values were located only on the brain area.

The measurement data was simulated from both phantoms in the same way as in the case of the homogeneous SoS. In the data simulation, the SoS distribution for each layer in the test and training set were drawn from the same range as mentioned above. That is, in each layer, each voxel had uniformly randomly a value from the SoS interval of that layer. Otherwise the generation of training and test sets was as in the previous simulations. In the reconstruction, a constant SoS of 1500m/s was used.

### 3.4 In vivo measurements

The approaches were tested with experimental data obtained from human hands using a photoacoustic scanner based on a Fabry–Pérot interferometer. In the measurements, different parts of the hands of several subjects were imaged. More details on the experimental setup and measurements can be found in Ref. [86].

With real data, gold standard reconstructions from a highly sampled complete data set are typically used as ground truth images. In this case, such reconstructions are not available, as the data is measured on one side of the object only. Thus, we use total variation (TV) constrained reconstructions [87] as our reference reconstructions. We note, that although these reconstructions are high-quality and represent our gold-standard, they do not completely correspond to the ground truth. Reference images were obtained by computing TV reconstructions using the SoS of 1585m/s. In the TV reconstruction, the regularisation parameter was chosen so that noise and regularisation artifacts were as minimal as possible. This was achieved by computing a set of reconstructions with small regularisation parameter and manual selection of best reconstructions by visual inspection. The measured data and corresponding computed reference images were divided to test and train set, so that train set included 25 samples and test set included 4 samples. This further training data limitation, compared to the simulated case, is natural for applications in 3D *in vivo* imaging, where high quality data is limitedly available. In addition, the sound speed of the medium was set to 1500m/s in the reconstructions.

## 4 Results and discussion

**Fig. 2:**
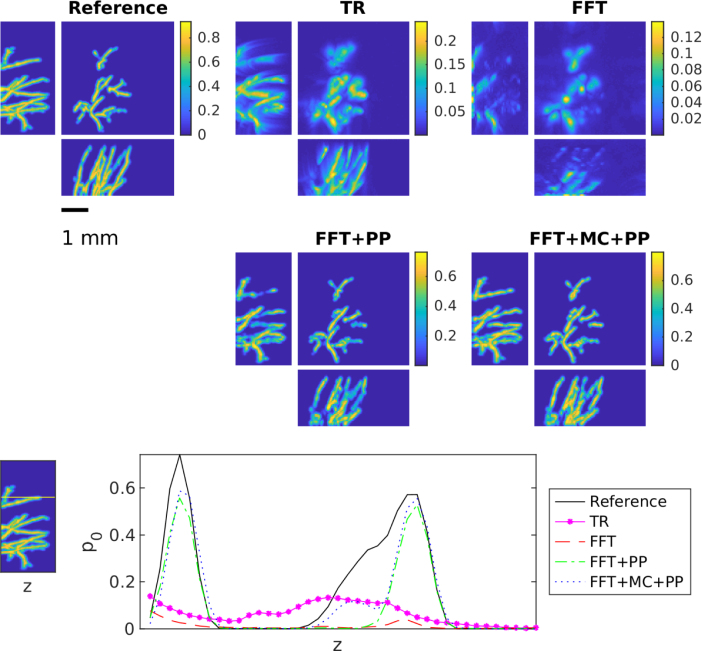
Maximum intensity projections of reconstructions obtained using time reversal (TR), FFT-based reconstruction method (FFT), FFT-based reconstruction method + post-processing (FFT+PP) and FFT-based reconstruction method with model correction + post-processing (FFT+MC+PP) in the case of the unknown homogeneous SoS. The SoS in the training was 1400m/s. Each reconstruction is presented in its own colourmap.

### 4.1 Homogenous speed of sound

**Fig. 3:**
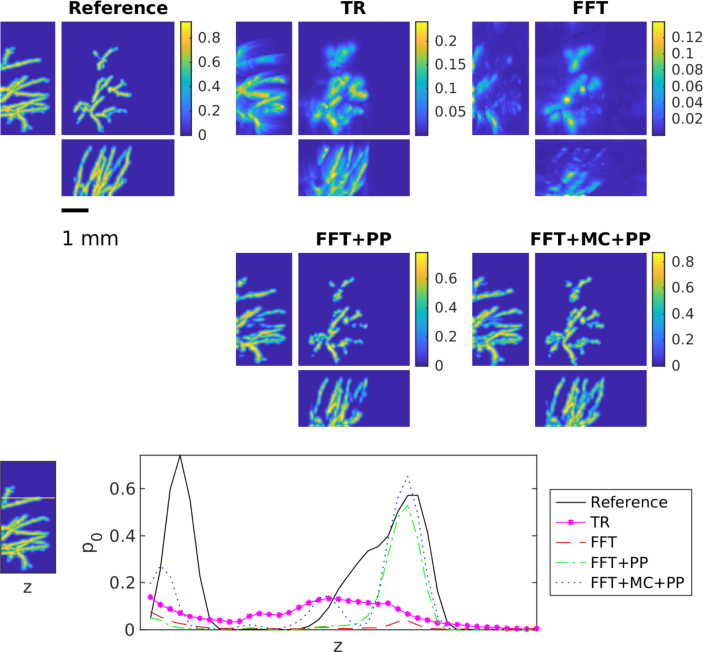
Maximum intensity projections of reconstruction obtained using time reversal (TR), FFT-based reconstruction method (FFT), FFT-based reconstruction method + post-processing (FFT+PP) and FFT-based reconstruction method with model correction + post-processing (FFT+MC+PP) in the case of the unknown homogeneous SoS. The SoS in the training was 1500m/s. Each reconstruction is presented in its own colourmap.

**Fig. 4:**
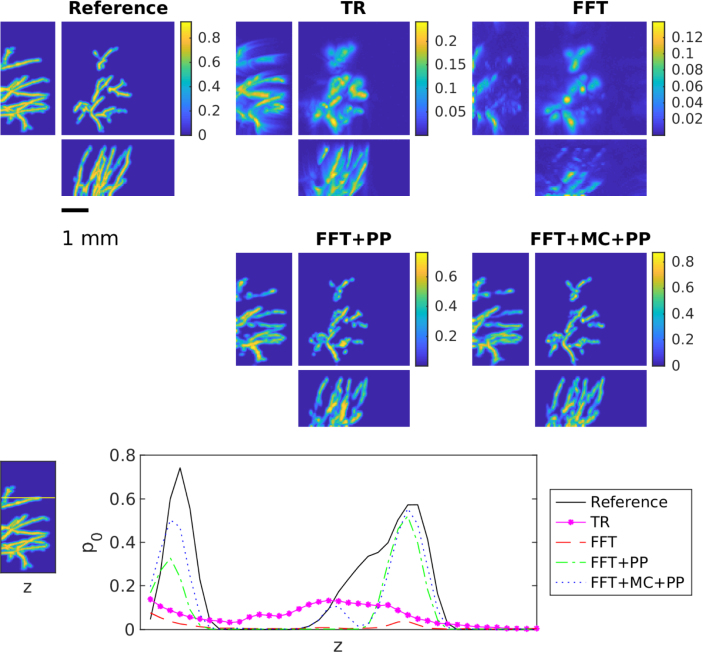
Maximum intensity projections of reconstruction obtained using time reversal (TR), FFT-based reconstruction method (FFT), FFT-based reconstruction method + post-processing (FFT+PP) and FFT-based reconstruction method with model correction + post-processing (FFT+MC+PP) in the case of the unknown homogeneous SoS. The SoS in the training was from range [1400,1600]m/s. Each reconstruction is presented in its own colourmap.

**Fig. 5:**
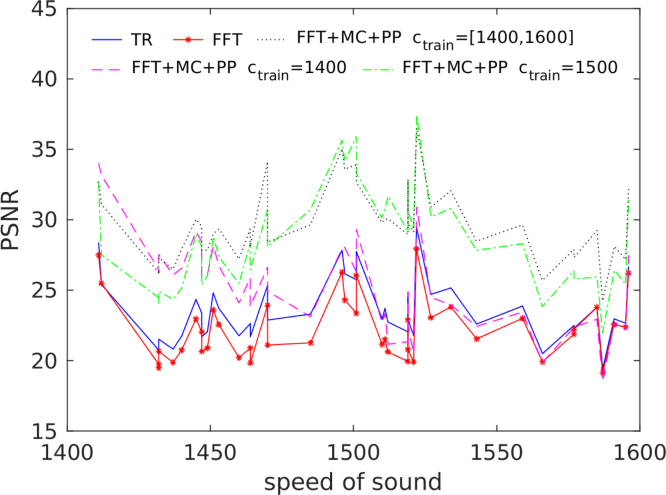
Peak signal to noise ratio (PSNR) as function of SoS for time reversal (TR), FFT-based reconstruction method (FFT) and FFT-based reconstruction method with model correction + post-processing (FFT+MC+PP) obtained using different SoS in training set in the case of the unknown homogeneous SoS.

The results obtained for the unknown SoS case are displayed in Figs. 2, 3 4, 5 and in Table 1. These reflect the different training scenarios of fixed SoS at 1400m/s in Fig. 2, 1500m/s in Fig. 3, and variable SoS between [1400,1600]m/s in Fig. 4, and a summary of errors is shown in Fig. 5. The time reversal and FFT-based reconstruction method produce similar reconstructions. Both of these reconstruction methods suffer as expected from limited-view and model mismatch errors due to wrong SoS used. Interestingly, the FFT-based reconstruction method exhibits more sensitivity to the mismatch in the chosen SoS values than time reversal. This can be seen especially clearly in the areas that are far from the sensor plane and in the vessels that are vertical to the sensor plane. In addition, quantitative values in these reconstructions are reduced. In contrast, learning-based reconstruction methods can improve both the quality and quantitative values of the reconstructions significantly. Nevertheless, the post-processing and model correction approach produce very similar reconstructions. However, overall results of model correction approach provides a slight improvement, especially in the areas were vessels were missing in the FFT reconstruction. Both learning-based methods can reduce limited-view and model mismatch errors successfully.

However, the corrective effect of these methods depends largely on the quality of the training data. The better the training data describes the object being imaged and the potential model mismatch (here by different SoS), the better the trained methods will be able to correct these errors. This is especially so for the modelling errors. This effect in illustrated in Fig. 5 and the error Table 1. When the correct SoS is close to 1400m/s, the best results are obtained using the training set were SoS was chosen only as 1400m/s. Correspondingly, if the correct SoS is close to 1500m/s, the training set with SoS of 1500m/s produces best results. However, if we consider all the samples in the test set where SoS varies between 1400 and 1600, then in general the best result is obtained with the test set that has SoS in range [1400,1600]m/s. Most notably, when the model correction network is only trained with 1400m/s, moving away from this ideal value degrades reconstruction quality significantly. In fact, when the network trained on 1400m/s is applied to values over 1500m/s the reconstruction quality becomes similar to time reversal. This underlines the importance of the training data to reflect the model mismatch. A similar trend can be seen in Table 1, when only one fixed SoS is used for the training model correction and post-processing only perform very similar, only when we consider the larger range of variable SoS an improvement can be observed of the model-correction and post-processing approach. This suggests a crucial role of the training data.

**Table 1:** The average peak signal to noise ratio (PSNR) and structural similarity index (SSIM) with standard deviation over the test set for time reversal (TR), FFT-based reconstruction method (FFT), FFT-based reconstruction method + post-processing (FFT+PP) and FFT-based reconstruction method with model correction + post-processing (FFT+MC+PP) obtained using different SoS distributions $c_{train}$ in training set in the case of the unknown homogeneous SoS data set.

$c_{train}\left(\mathrm{m/s}\right)$	TR	FFT	FFT+PP	FFT+MC+PP
1400				
PSNR	23.9±2.8	22.7±2.8	25.9±4.2	25.8±4.2
SSIM	0.75±0.10	0.72±0.12	0.90±0.06	0.90±0.06

1500				
PSNR	23.9±2.8	22.7±2.8	28.9±3.8	29.1±3.9
SSIM	0.75±0.10	0.72±0.12	0.95±0.04	0.95±0.03

[1400, 1600]				
PSNR	23.9±2.8	22.7±2.8	29.4±3.0	30.0±3.3
SSIM	0.75±0.10	0.72±0.12	0.95±0.03	0.96±0.02

### 4.2 Heterogeneous speed of sound

**Fig. 6:**
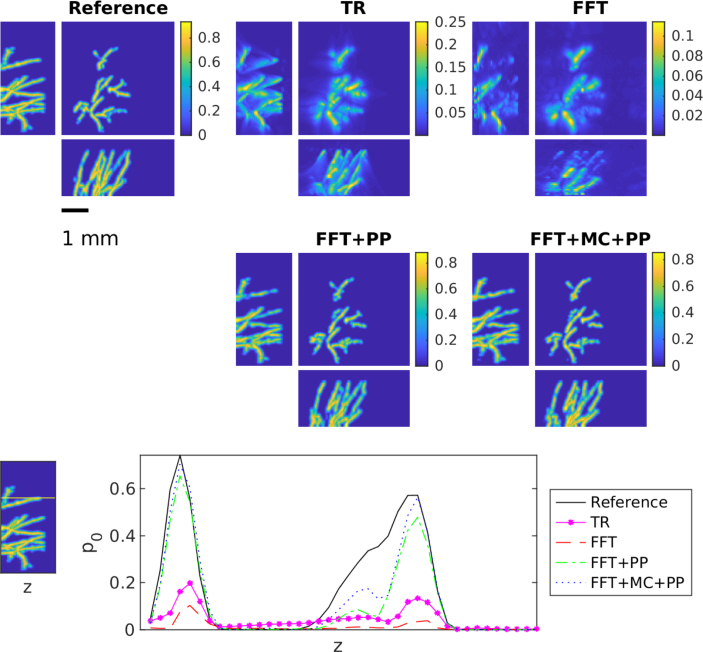
Maximum intensity projections of reconstruction obtained using time reversal (TR), FFT-based reconstruction method (FFT), FFT-based reconstruction method + post-processing (FFT+PP) and FFT-based reconstruction method with model correction + post-processing (FFT+MC+PP) in the case of the skin data set. Each reconstruction is presented in its own colourmap.

**Fig. 7:**
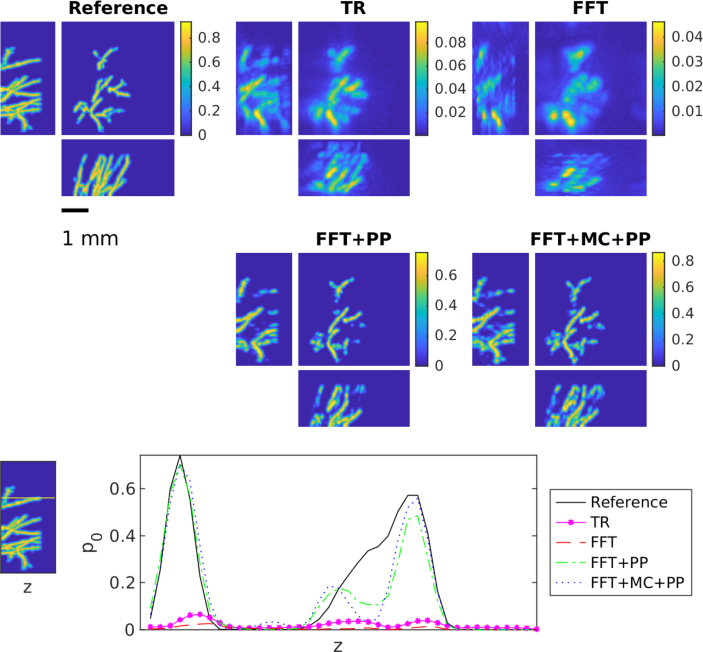
Maximum intensity projections of reconstruction obtained using time reversal (TR), FFT-based reconstruction method (FFT), FFT-based reconstruction method + post-processing (FFT+PP) and FFT-based reconstruction method with model correction + post-processing (FFT+MC+PP) in the case of the skull data set. Each reconstruction is presented in its own colourmap.

For the heterogeneous SoS, the results for the skin data set are shown in Fig. 6 and in Table 2. Correspondingly, Fig. 7 and Table 3 show the results for the skull data set. Both results are in line with the observations from the homogeneous but unknown SoS described above. The FFT-based reconstruction method gives the worst reconstruction that suffers from strong errors and hence can be highly improved with the learning-based methods. The results of the learned methods are very similar and difference between these two approach is even smaller than in the case of the unknown SoS. In the case of the skin data sets, modelling errors are quite small and both learned approaches can correct these errors successfully. However, in the case of the skull data set, the effect of modelling errors is emphasised. Interestingly in this case, the learned methods both cannot correct the error as successfully as in the case of the skin data even though the SoS is modelled properly in the training set. This seems to be a limiting case and further research to compensate such strong mismatch is needed.

**Table 2:** The average peak signal to noise ratio (PSNR) and structural similarity index (SSIM) with standard deviation over the test set for time reversal (TR), FFT-based reconstruction method (FFT), FFT-based reconstruction method + post-processing (FFT+PP) and FFT-based reconstruction method with model correction + post-processing (FFT+MC+PP) in the case of the skin dataset.

Method	PSNR	SSIM
TR	23.6±2.7	0.77±0.09
FFT	22.9±2.8	0.74±0.11
FFT+PP	32.8±3.0	0.98±0.01
FFT+MC+PP	32.4±3.1	0.98±0.01

**Table 3:** The average peak signal to noise ratio (PSNR) and structural similarity index (SSIM) with standard deviation over the test set for time reversal (TR), FFT-based reconstruction method (FFT), FFT-based reconstruction method + post-processing (FFT+PP) and FFT-based reconstruction method with model correction + post-processing (FFT+MC+PP) in the case of the skull dataset.

Method	PSNR	SSIM
TR	23.6±2.9	0.77±0.10
FFT	23.2±3.0	0.78±0.10
FFT+PP	29.0±3.1	0.95±0.02
FFT+MC+PP	29.0±3.1	0.95±0.02

**Fig. 8:**
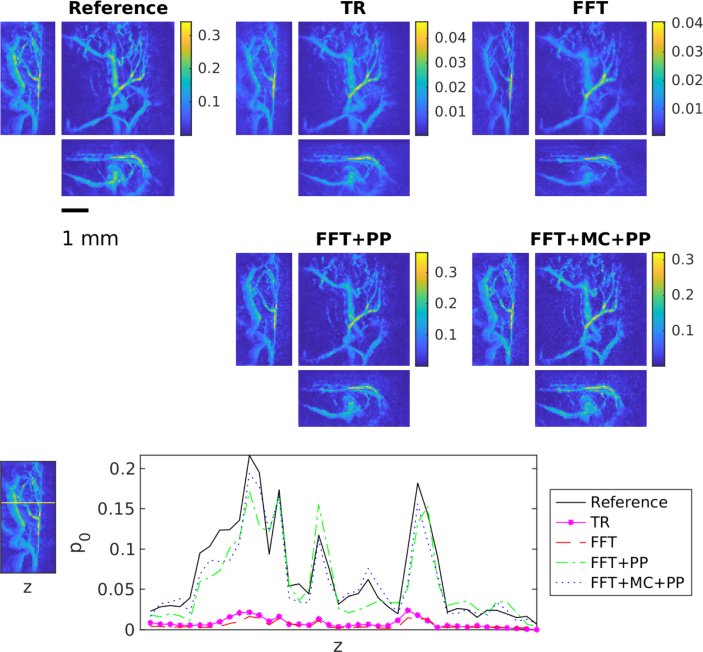
Maximum intensity projections of reconstruction obtained using time reversal (TR), FFT-based reconstruction method (FFT), FFT-based reconstruction method + post-processing (FFT+PP) and FFT-based reconstruction method with model correction + post-processing (FFT+MC+PP) in the case of the *in vivo* measurements. Each reconstruction is presented in its own colourmap.

**Table 4:** The average peak signal to noise ratio (PSNR) and structural similarity index (SSIM) with standard deviation over the test set for time reversal (TR), FFT-based reconstruction method (FFT), FFT-based reconstruction method + post-processing (FFT+PP) and FFT-based reconstruction method with model correction + post-processing (FFT+MC+PP) in the case of the *in vivo* measurements.

Method	PSNR	SSIM
TR	32.0±2.5	0.839±0.006
FFT	31.8±2.5	0.835±0.008
FFT+PP	36.6±2.5	0.925±0.006
FFT+MC+PP	36.8±2.4	0.926±0.006

### 4.3 In vivo measurements

The results for *in vivo* measurements are presented in Fig. 8 and Table 4. Also the result of the *in vivo* measurements conform with the previous results. We can obtain clear improvement in the reconstructions by using either of the learning-based reconstruction methods. We can see, that the reconstructions obtained by the post-processing and model correction approach are visually very similar and the model correction does provide only a slight improvement in quantitative measures. This improvement could be explained by the cross-section in Fig. 8, where the post-processing approach seems to under or overshoot the absorption values in the vessels and profile of the model correction approach is closer to the absorption values from the reference reconstruction. Nevertheless, for the experimental data the model correction could not provide a significant improvement over post-processing. Finally, in the training, a slower convergence of training error has been observed, which may be due to smaller training data size.

### 4.4 Extensions and limitations

Overall, we can conclude that the examined learning-based methods are powerful for correcting limited-view artefacts and modelling errors due to SoS variations. The post-processing alone already provides good results but with the model correction we can gain slight improvements both in quality with respect to recovered quantitative values. Nevertheless, most of the improvements are dependent on the right choice of training data. However, improvements gained with the model correction come with a cost. Training the model-corrected approach is approximately four times slower than training of just the post-processing due to an expensive network in the complex *k*-space. In addition, we also noticed that training of the model correction approach is harder, in the sense that longer training is needed for convergence and to provide good generalisation to the test set. This may be because of the training in the model correction is done in *k*-space and small changes in *k*-space can have large impact in image space. Other reasons could be the limited size of the training sets, especially in the case of the *in vivo* measurements, which is a common limitation for applications of learned methods to *in vivo* measurements. The potential influence of training data size may be a thus an interesting area for future investigation.

In the model correction approach, we used a quite simple network architecture, as we postulated that only local changes are needed and hence a large receptive field is not required. To confirm this, we also tested a complex U-Net, with same architecture as for the post-processing, and indeed no significant improvements were observed. It is worth mentioning that we have also examined the performance of the model correction alone without a post-processing network, where small improvements can be observed, but the network in *k*-space alone did not manage to correct for the limited-view artefacts, thus the performance gain was not significant enough. In the future, different networks could be investigated to see if one could improve the performance of the model correction approach. For this purpose one would need to train a network to interpolate the missing *k*-space data to compensate for the limited-view geometry. We can take inspiration here from research in magnetic resonance imaging. For instance complex valued generative adversarial networks [88], multi-scale residual networks using dual frame U-Net [89] and complex dense fully convolutional networks [90] have been used to process the *k*-space data. However, increased complexity of the model correction network also rapidly increases memory requirements.

Finally, we have observed when introducing an additional component that is designated to correct for modelling errors the choice of training data becomes even more crucial. We have observed that results are more sensitive to the information encoded in the training data, as the model correction component needs to be able to learn a good correction procedure. In other words, more complexity in the model needs to be properly reflected in the choice of training data.

## 5 Conclusions

We have studied the possibility of compensating for modelling errors given by a mismatch of SoS distributions in fast FFT-based reconstructions for limited-view 3D photoacoustic tomography. We proposed a model-correction term in *k*-space to compensate for wrong SoS that can be trained jointly with a post-processing network to improve image quality and compensate for limited-view artefacts. Additionally, we have examined the influence of the training data on the task to compensate for modelling errors.

We have shown that one can successfully compensate for model errors due to wrong SoS choices, by both a model-correction term and post-processing alone, if the training data provides a good representation of the errors that need to compensated. In particular, a learnable correction does require a carefully designed training data to train the introduced correction term and the performance is dependent on a good choice of training data.

In conclusion, based on the presented results, first and foremost a careful choice of training data is of great importance when correcting modelling errors in a supervised training. The training data should not only give a good representation of the relevant structures in the image domain but also well resemble potential modelling errors that can occur in the imaging problem.

## Declaration of competing interest

The authors declare that they have no known competing financial interests or personal relationships that could have appeared to influence the work reported in this paper.

## Data Availability

Codes are available at https://github.com/jpoimala/learnedFastPAT.

## Footnotes

1 https://github.com/jpoimala/learnedFastPAT.

2 https://github.com/wavefrontshaping/complexPyTorch.
